# Smokeless tobacco use: its prevalence and relationships with dental symptoms, nutritional status and blood pressure among rural women in Burkina Faso

**DOI:** 10.1186/s12889-020-08700-x

**Published:** 2020-04-28

**Authors:** Jeoffray Diendéré, Augustin Nawidimbasba Zeba, Léon Nikièma, Ahmed Kaboré, Paul Windinpsidi Savadogo, Somnoma Jean-Baptiste Tougma, Halidou Tinto, Arouna Ouédraogo

**Affiliations:** 1 Public Health Department, Research Institute for Health Sciences, 399, Avenue de la Liberté, 01 BP 545, Bobo-Dioulasso, 01 Burkina Faso; 2 Public Health Department, Centre Muraz, Bobo-Dioulasso, Burkina Faso; 3 Joseph Ki-Zerbo University, BP 5705, Ouagadougou, 01 Burkina Faso; 4 Institute of Environment and Agricultural Research (INERA/CNRST), rue Guisga, 04 BP 8645, Ouagadougou, Burkina Faso; 5 Sourô Sanou University Hospital, 01 BP 676, Bobo-Dioulasso, Burkina Faso; 6 Clinical Research Unit of Research Institute for Health Sciences Nanoro, 11 BP 218, Ouagadougou, Burkina Faso; 7 Department of Psychiatry, Yalgado Ouédraogo University Hospital, Joseph Ki-Zerbo University, 01 BP 5705, Ouagadougou, Burkina Faso

**Keywords:** Smokeless tobacco, Alcohol co-use, Dental symptoms, Undernourishment, Blood pressure, Rural women, Burkina Faso

## Abstract

**Background:**

Sub-Saharan women use smokeless tobacco (SLT) more than smoked tobacco. Among Western African countries, the estimated weighted prevalence of SLT use in rural women was found to be the highest in Burkina Faso (after Sierra Leone). This study aimed to assess the prevalence of SLT use and its associated factors among rural women in Burkina Faso by using nationally representative data.

**Methods:**

We used data from the 2013 STEPwise approach to Surveillance (STEPS) study, which provided sociodemographic, clinical (anthropometric, systolic blood pressure [SBP], diastolic blood pressure [DBP] and dental symptoms), biological (total and high-density lipoprotein cholesterol and fasting blood sugar), and tobacco and alcohol consumption data. Data for 1730 rural women were used, and we performed Student’s chi-squared and logistic regression analyses.

**Results:**

The prevalence of current SLT use was 13.8% (95% CI: 12.2–15.5). Significant risks for SLT use were the presence of dental symptoms (adjusted odds ratio [aOR] = 2.59; *p* < 0.001), undernourishment (aOR = 1.78; *p* < 0.01), decreased waist circumference (aOR = 0.98; *p* < 0.05), decreased DBP (aOR = 0.97; *p* < 0.01), increased SBP (aOR = 1.01; *p* < 0.05), and increased differential blood pressure (aOR = 1.01; *p* < 0.05). The co-use of alcohol was also a significant risk factor (aOR = 2.80; *p* < 0.001).

**Conclusion:**

The prevalence of current SLT use was high among rural women in Burkina Faso, and significant concerns for users included alcohol co-use, the occurrence of dental symptoms, undernourishment, and an increase in differential blood pressure. National Public Health interventions are needed to reduce SLT use and its health-related concerns.

## Background

Smokeless tobacco (SLT) and smoked tobacco (ST) are both used in sub-Saharan African (SSA) countries, and SLT is used more than twice as often in rural areas than in urban areas [1]. The prevalence of SLT use in women is more than three times higher in rural areas than in urban areas [1]. In SSA countries, the most favoured form of tobacco for females is SLT, mostly used orally in a chewable form [2, 3]. In Qatar, women have impaired oral health approximately twice as frequently as men do, and SLT use is approximately four times higher when people have impaired oral health [4], which is sometimes considered to be a motivation for using SLT. The level of awareness of the health risks associated with the consumption of SLT products is low, particularly among people with low socioeconomic status or those in rural areas [5]. The sociocultural environment is an important influencing factor in the initiation of SLT use [6]. In a report on 10 West African countries between 2008 and 2012, the weighted prevalence estimate of SLT use in rural women was highest in Burkina Faso (after Sierra Leone), and more than 80% of the women were reported to use chewing tobacco [1]. Compared to the use of ST, the use of SLT (especially chewing tobacco) by females is not socially stigmatizing in Burkina Faso. The harmfulness of SLT appears to be misunderstood, and traditionally, symbolic gifts (from urban residents) to elders in rural areas often include tobacco products. National Public Health interventions regarding tobacco use exclusively target ST consumption, devoting only limited attention to other types of products, including SLT. However, the toxicity, mutagenicity, and carcinogenic effects of the hazardous chemicals present in SLT products have been documented [7]. Similar to ST, SLT includes nicotine as the primary psychoactive and addictive molecule, and the systemic absorption and nicotine levels were similar in SLT users and cigarette smokers [8]. In addition, depending on the cultivated soils and production processes, some unusually high levels of known components and unexpected toxins have been identified in SLT leaves [9, 10]. Recent international studies revealed serious health concerns related to SLT consumption, specifically oral health impairment [11, 12], nutritional disorders [13], co-use of psychoactive substances, and cardiovascular disease [14–16]. In Burkina Faso, no previous study using a representative sample has reported the national prevalence of SLT use and its health-related consequences in rural areas, particularly among females. The first national survey using the World Health Organization (WHO) standardized STEPwise approach to Surveillance (STEPS) [17] also provided data on tobacco consumption. Our study aimed to assess the prevalence of SLT use and its relationships with dental symptoms, nutritional status, and blood pressure level among rural women in Burkina Faso using nationally representative data.

## Methods

**Fig. 1:**
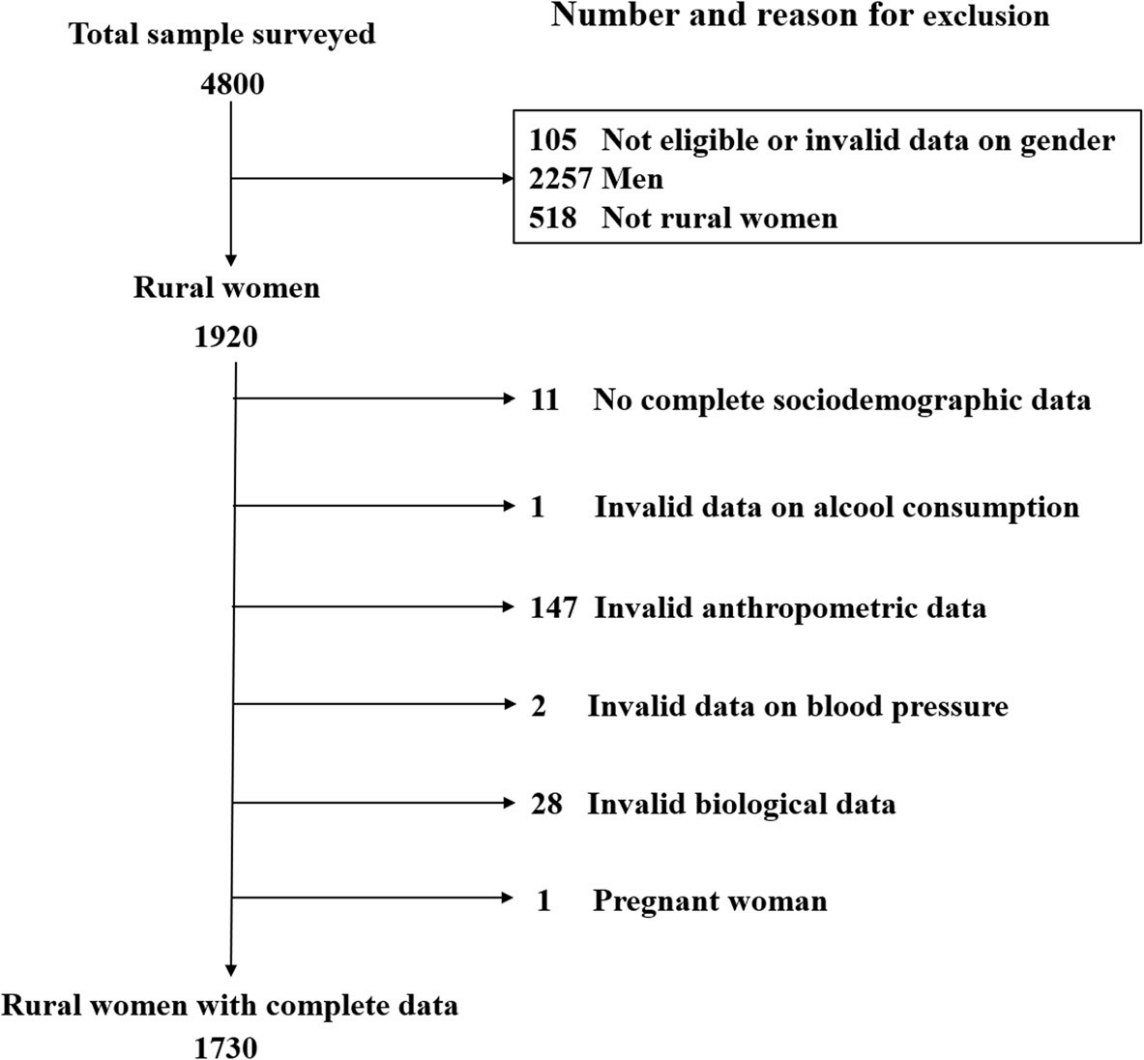
Flow diagram of the women included in the analyses

We used data from the first Burkina Faso national survey conducted in 2013, which was based on the WHO STEPS methodology [17]. The protocol of the STEPS survey was approved by the Ethics Committee for Health Research of the Ministry of Health of Burkina Faso (deliberation No: 2012-12092; December 05, 2012). Written informed consent was systematically obtained from each participant in the STEPS survey. The sample calculation and data collection methods have been described [18, 19]. The number of surveyed rural women was 1920 and we reanalysed variables for those with complete data for the sociodemographic, lifestyle, nutritional and biological parameters and with responses to items that screened dental symptoms. Thus, we included 1730 women in the analyses, and Fig. 1 presents the diagram flow of the women we studied.

### Variables of interest

Participants’ demographic variables included age, marital status, education level, and occupation. We also reported whether the women were living in households with at least one member aged ≥18 years. The anthropometric characteristics were weight (kg), height (m), body mass index (BMI = weight/height^2^, in kg/m^2^; a BMI < 18.5 kg/m^2^ was considered underweight, a BMI of 18.5–24.9 kg/m^2^ was considered normal weight, and a BMI ≥ 25 kg/m^2^ was considered overweight/obese) and waist circumference (cm). The biological characteristics tested in blood were total cholesterol (mmol/l), high-density lipoprotein cholesterol (HDL, in mmol/l), and fasting sugar (mmol/l). Blood pressure (in mmHg, which included systolic blood pressure [SBP] and diastolic blood pressure [DBP] values) was measured three times, and we kept only the mean value for each indicator; levels ≥140/90 mmHg defined high blood pressure (HBP), and the difference between the mean values of SBP and DBP (SBP - DBP) was used to determine the differential blood pressure. Lifestyle factors were determined by using self-reporting techniques and included current (past month) alcohol consumption and current (past year) smokeless (chewing, snorting) or smoked tobacco consumption. Dental problems were also recorded by using a self-reported method to report having the following during the past 12 months: i) difficulties chewing food, ii) difficulties with speech/trouble pronouncing word or iii) pain or discomfort in the teeth/mouth.

### Statistical analyses

We used StataCorp Stata Statistical Software for Windows (Version 12.0, College Station, Texas, US) to analyse the data. The quantitative variables were expressed as the means ± standard deviations, and the categorical variables were expressed as percentages (%), with a confidence interval of 95% (95%, CI). Student’s t-test was used to compare quantitative variables, and the chi-squared test was used for categorical variables. Logistic regression analysis was performed to identify the clinical, biological, and lifestyle factors associated with SLT after adjusting for sociodemographic features. The model was determined by backward elimination (i.e., the progressive elimination of nonsignificant factors by decreasing order of significance). For all analyses, a *p*-value below 0.05 was considered significant.

## Results

The mean age in the sample was 37.8 ± 10.9 years, and other sociodemographic characteristics are presented in Table 1. Only one woman used ST (> 0.0%). The prevalence of SLT use was 13.8% (95% CI: 12.2–15.5), the prevalence of current alcohol use was 24.6% (95% CI: 22.6–26.7), the prevalence of dental symptoms was 24.1% (95%; CI: 22.1–26.2), the prevalence of undernourishment was 16.0% (95%; CI: 14.3–17.8), and the prevalence of HBP was 14.6% (95%; CI: 13.0–16.4). The means of all anthropometric parameters for SLT users were significantly lower than those for nonusers: 53.2 ± 9.0 kg vs 56.9 ± 10.3 for weight, 20.2 ± 3.1 kg/m^2^ vs 21.7 ± 3.6 for BMI and 74.6 ± 10.7 cm vs 76.4 ± 11.7 for waist circumference (each *p*-value< 0.05), respectively. The mean SBP was significantly higher in SLT users than in nonusers (122.9 ± 19.8 mmHg vs 119.6 ± 16.1; *p* < 0.01), as was the differential blood pressure (46.3 ± 13.9 mmHg vs 42.8 ± 11.3; *p* < 0.001). However, there was no significant difference in the mean DBP between SLT users and nonusers (76.6 ± 10.3 mmHg vs. 76.8 ± 10.0; *p* = 0.72). The mean glycaemia was also higher for SLT users (4.1 ± 1.5 mmol/l vs. 3.8 ± 1.5 for nonusers, *p* < 0.05; Table 2).

**Table 1:** Sociodemographic characteristics for all rural women (*n* = 1730)

	Overall *n* = 1730		Not users of smokeless tobacco *n* = 1492		Users of smokeless tobacco *n* = 238		
	n	% (95% CI)	n	% (95% CI)	n	% (95% CI)	*p* value
Age mean ± standard deviation (years)	1730	37.8 ± 10.9		36.7 ± 10.5		44.6 ± 11.0	***
Age range (years)							***
- 25–34	809	46.8 (44.4–49.1)	757	50.7 (48.2–53.3)	52	21.8 (16.8–27.6)	
- 35–49	592	34.2 (32.0–36.5)	503	33.7 (31.3–36.2)	89	37.4 (31.2–43.9)	
- > 49	329	19.0 (17.2–20.9)	232	15.6 (13.7–17.5)	97	40.8 (34.5–47.3)	
Marital status							***
- Married/cohabitating	1553	89.8 (88.2–91.2)	1357	91.0 (89.4–92.4)	196	82.4 (76.9–87.0)	
- Singles	177	10.2 (8.8–11.8)	135	9.0 (7.6–10.6)	42	17.6 (13.0–23.1)	
Education level							***
- No formal education	1558	90.1 (88.6–91.4)	1326	88.9 (87.2–90.4)	232	97.5 (94.6–99.1)	
- Primary school or more	172	9.9 (8.6–11.4)	166	11.1 (9.6–12.8)	6	2.5 (0.9–5.4)	
Occupation							NS
- Employed/Self-employed	1002	57.9 (55.6–60.3)	866	58.0 (55.5–60.6)	136	57.1 (50.6–63.5)	
- Others (Students+household-keepers+unemployed)	728	42.1 (39.7–44.4)	626	42.0 (39.4–44.5)	102	42.9 (36.5–49.4)	
Having at least one family member aged≥18 years, yes	1286	74.3 (72.2–76.4)	1118	74.9 (72.7–77.1)	168	70.6 (64.4–76.3)	NS
Current alcohol use, yes	425	24.6 (22.6–26.7)	316	21.2 (19.1–23.3)	109	45.8 (39.3–52.4)	***
Presence of dental symptom, yes	417	24.1 (22.1–26.2)	312	20.9 (18.9–23.1)	105	44.1 (37.7–50.7)	***

*NS* indicates Non-Significant *p* value; *** indicates *p* value < 0.001

Only one woman used the ST (> 0.0%; 95% CI: > 0.0–0.03)

**Table 2:** Nutritional, clinical, and biological features of rural women according to the use of psychoactive substances (*n* = 1730)

Parameters	Mean ± standard deviation or % (95% confident interval)						
	Overall	Current SLT users	Not current SLT users	*p* value	Current SLT and alcohol users	Current SLT and not alcohol users	*p* value
	(*n* = 1730)	(*n* = 238)	(*n* = 1492)		(*n* = 109)	(*n* = 1621)	
Waist circumference,(cm)	76.2 ± 11.6	74.6 ± 10.7	76.4 ± 11.7	*	75.0 ± 11.8	76.3 ± 11.6	NS
Weight,(kg)	56.4 ± 10.2	53.2 ± 9.0	56.9 ± 10.3	***	52.7 ± 8.1	56.7 ± 10.3	***
Body mass index,(kg/m^2^)	21.5 ± 3.6	20.2 ± 3.1	21.7 ± 3.6	***	20.2 ± 2.8	21.6 ± 3.6	***
Nutritional state							
- Underweight	16.0 (14.3–17.8)	28.6 (22.9–34.8)	13.9 (12.2–15.8)	***	28.4 (20.2–37.9)	15.1 (13.4–17.0)	**
- Normal	70.9 (68.7–73.1)	66.0 (59.6–72.0)	71.7 (69.4–74.0)	NS	66.1 (56.4–74.9)	71.3 (69.0–73.4)	NS
- Overweight/obesity	13.1 (11.6–14.8)	5.5 (2.9–9.2)	14.4 (12.6–16.2)	***	5.5 (2.0–11.6)	13.6 (12.0–15.4)	***
Systolic blood pressure (mmHg)	120.0 ± 16.7	122.9 ± 19.8	119.6 ± 16.1	**	119.9 ± 16.3	120.1 ± 16.7	NS
Diastolic blood pressure (mmHg)	76.8 ± 10.0	76.6 ± 10.3	76.8 ± 10.0	NS	76.3 ± 9.8	76.8 ± 10.05	NS
Differential blood pressure (mmHg)	43.3 ± 11.8	46.3 ± 13.9	42.8 ± 11.3	***	43.6 ± 11.3	43.2 ± 11.8	NS
High blood pressure, yes (> 140/90 mmHg)	14.6 (13.0–16.4)	16.4 (11.9–21.7)	14.3 (12.6–16.2)	NS	13.8 (7.9–21.7)	14.7 (13.0–16.5)	NS
Blood sugar(mmol/l)	3.9 ± 1.5	4.1 ± 1.5	3.8 ± 1.5	*	4.2 ± 1.4	3.9 ± 1.5	*
Hyperglycemia, yes (> 6.1 mmol/l)	5.0 (4.0–6.1)	5.5 (2.9–9.2)	4.9 (3.9–6.1)	NS	5.5 (2.0–11.6)	4.6 (3.6–5.7)	NS
HDL cholesterol,(mmo/l)	0.9 (0.5)	0.9 ± 0.4	0.9 ± 0.5	NS	1.0 ± 0.5	0.9 ± 0.5	NS
Total cholesterol,(mmol/l)	3.1 (0.8)	3.1 ± 0.8	3.1 ± 0.8	NS	3.2 ± 1.0	3.0 ± 0.8	*

*NS* indicates Non-Significant *p* value; * indicates *p* value < 0.05; ** indicates *p* value < 0.01; *** indicates *p* value < 0.001

The results of the logistic regression are reported in Table 3. Significant risks associated with SLT use were the co-use of alcohol (adjusted odds ratio [aOR] = 2.80; 95% CI: 2.06–3.80), the presence of dental symptoms (aOR = 2.59; 95% CI: 1.91–3.51), undernourishment (aOR = 1.78; 95% CI: 1.24–2.55), a decreased waist circumference (aOR = 0.98; 95% CI: 0.97- < 1.00; *p* < 0.05), decreased DBP (aOR = 0.97; 95% CI: 0.95–0.99) and increased SBP (aOR = 1.01; 95% CI: > 1.00–1.03; *p* < 0.05). When replacing both the SBP and DBP variables with the single differential blood pressure (SBP-DBP in mmHg) in the multivariable model, the differential unit increase was also significant (aOR = 1.01; 95% CI: > 1.00–1.02; *p* < 0.05). Concerning sociodemographic variables, significant risk factors were old age (with the 25–34-year-old subgroup as the reference; aOR = 2.14; 95% CI: 1.46–3.13 in the 35–49-year-old subgroup; aOR = 4.31; 95% CI: 2.86–6.48 in the > 49-year-old subgroup) and lack of education (with the group with a secondary education level as the reference; aOR = 3.02; 95% CI: 1.28–7.10).

**Table 3:** Factors associated with smokeless tobacco use among rural women in Burkina Faso (*n* = 1730)

Factors	Univariate analysis			Multivariate analysis		
	cOR	95% CI	p value	aOR	95% CI	***p*** value
Age range (years)						
- 25–34	1					
- 35–49	1.58	1.80–3.69	***	2.14	1.46–3.13	***
- > 49	6.09	4.21–8.79	***	4.31	2.86–6.48	***
Occupation: others#, vs employed/self-employed	1.04	0.79–1.37	NS	0.97	0.71–1.32	NS
Marital status: Singles vs married/cohabiting (ref)	2.15	1.48–3.14	***	1.12	0.73–1.74	NS
Having at least one family member aged ≥18 years: No, vs yes (ref)	1.25	0.92–1.69	NS	0.89	0.61–1.26	NS
*Education level: no education or primary school vs secondary or more (ref)	4.84	2.12–11.06	***	3.02	1.28–7.10	*
Current alcohol use: yes, vs no (ref.)	3.14	2.37–4.18	***	2.80	2.06–3.80	***
Presence of dental symptom: yes, vs no (ref)	2.99	2.25–3.97	***	2.59	1.91–3.51	***
Waist circumference (cm)	0.98	0.97–0.99	*	0.98	0.97- < 1.00	*
Undernourishment (BMI < 18.5 kg/m^2^): yes, vs no (ref)	2.47	1.80–3.39	***	1.78	1.24–2.55	**
Systolic blood pressure (mmHg)	1.01	> 1.00–1.02	**	1.01	> 1.00–1.03	*
Diastolic blood pressure (mmHg)	> 0.99	0.98–1.01	NS	0.97	0.95–0.99	**
Blood sugar (mmol/L)	1.10	1.01–1.20	*	1.06	0.96–1.18	NS
HDL cholesterol (mmol/l)	1.08	0.81–1.43	NS	0.90	0.64–1.25	NS
Total cholesterol (mmol/L)	1.05	0.88–1.24	NS	0.97	0.78–1.21	NS

*NS* indicates Non-Significant *p* value; * indicates *p* value < 0.05; ** indicates *p* value < 0.01; *** indicates *p* value < 0.001

*cOR* crude odds ratio, *aOR* adjusted odds ratio, *95% CI* 95% confidence interval, others#: included professions with inconstant incomes (students, homemakers, and the unemployed). In this multivariate model, by replacing the dichotomic variable “undernourishment: yes or no” with the quantitative variable “BMI in kg/m^2^” and the two quantitative variables SBP and DBP with the differential blood pressure (SBP-DBP in mmHg), we observed that the decrease in the unit of BMI (aOR = 0.88; 95% CI: 0.83–0.93; *p* < 0.001) or the increase in the unit of differential blood pressure (aOR = 1.01; 95% CI: > 1.00–1.02; *p* < 0.05) were significantly associated with SLT use

## Discussion

The prevalence of current SLT use was high among rural women in Burkina Faso, and given the current state of epidemiologic transition, the relationship of SLT use with noncommunicable diseases should make SLT use a public health concern.

### Prevalence of SLT use

The prevalence of current SLT use among rural women in Burkina Faso was 13.8% (95% CI: 12.2–15.5), while only one woman used ST. Such statistics suggest a socially permissive attitude towards SLT use by females. A lower standardized prevalence estimate of 3.86% (95% CI: 3.22–4.48) was noted 2 years earlier (in 2011 and only in the sample of women aged 15–49 years) [1]. A current prevalence of SLT use of 17.4% (95% CI: 14.5–20.5) was found in rural women in three Ethiopian pastoral communities in which SLT chewing was a longstanding tradition [6], and the current prevalence of SLT use was 10.1% (95% CI: 8.8–11.4) in Mozambique [20]. Studies focusing on female SLT consumption with details for subgroups of rural and urban women are scarce in SSA, especially in West Africa. However, 3.3% of women and 4.7% of rural residents in Kenya were reported to be SLT users [21], as were 2.8% of women in Uganda [3]. Produced using a traditional technique, SLT is equivalent to a local psychoactive product and is widely available in rural Burkina Faso. There is no inventory concerning local psychoactive substances in Burkina Faso, and SLT seemed to be among the favourites of rural Burkinabé women, along with the roots of *Sarcocephalus latifolius* (a plant that provides synthetic tramadol), or non-medical tramadol, in Cameroon [22, 23] and khat/miraa in rural Kenya [24, 25].

### Co-use of alcohol

Current SLT users were also frequently current alcohol users (aOR = 2.80; 95% CI: 2.06–3.80), as has been reported for women in Cambodia (aOR = 1.49; 95% CI: 1.12–1.98) [26] and in Kenya (aOR = 2.58; *p* = 0.001 for those with alcohol experience; aOR = 4.84; *p* = 0.007 for episodic binge drinkers) [21]. SLT and alcohol are both psychoactive substances containing nicotine and ethanol molecules, respectively [27], and their synergistic interactive effects potentiate physical and psychological pleasure [28]. Therefore, polyconsumption of both tobacco and alcohol was not surprising [29]. Unfortunately, the use of tobacco and alcohol are considered major cardiovascular risk factors. Drinking alcohol is known to increase both HDL-C and total cholesterol concentrations [30], and the concurrent use of tobacco and alcohol enhances these increases [31]. Although the mean HDL cholesterol and total cholesterol values were not higher in SLT users than in nonusers, the mean total cholesterol value was significantly higher in concurrent current SLT and alcohol users than in nonusers (Table 2).

### Blood pressure and SLT use

SLT use was associated with increases in SBP (aOR = 1.01; *p* < 0.05) and differential blood pressure (aOR = 1.01; *p* < 0.05) (Table 3). Substantial nicotine is absorbed from SLT products [14]. The predominant cardiovascular effects of nicotine result from the activation of the sympathetic nervous system, which causes a hypertensive effect [32]; the increase in SBP in our sample (aOR = 1.01; *p* < 0.05) was consistent with this effect and was consistent with the findings of Onwuchekwa in rural Niger (aOR = 2.32; *p* < 0.05 among rural residents) [33]. Furthermore, age-related increases in SBP have been reported to be greater in women than men [34, 35]; thus, the increase in SBP was more perceptible in our sample of women than it might have been in a sample of both men and women. However, we observed a decrease in DBP with age (0.97; *p* < 0.01) (Table 3) in SLT users. We should note that there was an increase in DBP level with age from an average of 78.4 (±9.5) mmHg at approximately 36 years of age to 83.1 (±11.8) mmHg at 53 years and then a decrease to 73.4 (±10.1) mmHg at 69 years of age [36]. An inverse association of DBP and age has been well established [36], and it may partly explain our finding. Nicotine interacts with central oestrogenic pathways [37], which may help explain the non-homogenous effects of nicotine on SBP and DBP. SLT is usually locally produced using a non-standardized processed, and tobacco is grown in different types of soils in which mineralogic contents (such as sodium) may affect blood pressure in users [38]. In addition, the use of pesticides in rural Burkina Faso may cause soil contamination by staining tobacco leaves [39], which has been implicated in BP increases [40]. The effects of chronic consumption of kola nuts on the cardiovascular system should be considered [41, 42]. Research showed that one in two adults used kola nuts in Burkina Faso [43], usually under similar conditions (i.e., dental health impairment conditions [44]). Unspecified effects of interactions between SLT and kola nut products on BP may be possible. In short, cardiovascular disturbance in SLT users was evident in the substantial increase in differential blood pressure (aOR = 1.01; *p* = 0.041; Table 3).

### Dental symptoms

SLT use was associated with the presence of some dental symptoms (aOR = 2.59; 95% CI: 1.91–3.51). Cheema et al. reported an association with poor oral status (aOR = 3.90; 95% CI: 1.75–8.69) in Qatar [4]. Users of this psychoactive substance (SLT) develop various expectancies for using it according to the different effects generated by its consumption depending on the context. Oro-dental pain and burning mouth syndrome are common in SSA countries, and poor oral health service utilization has been reported in these countries [45, 46]. The nicotine delivered by SLT products increases sensory irritation [47], and because dental care is not available in rural Burkina Faso [44], rural women are likely to use SLT for the pain or discomfort associated with dental symptoms. Because chewing food might exacerbate dental pain, in the absence of treatment, those with dental symptoms use SLT to locally anaesthetize teeth or the oral cavity to be able to eat without pain. Such behaviour was noted in a supplemental qualitative study (interview) in three Ethiopian pastoral communities with a long tradition of SLT chewing [6]. Furthermore, psychoactive substance consumption resulting from addictive behaviours includes gestural rituals [48] that have been established over time. Tooth and periodontal damage was found to be common in SLT users [49], mainly female chewers [50], and the intention to use SLT for dental pain suppression could establish a vicious circle between dental symptoms and the repeated and inefficient application of SLT as treatment.

### Undernourishment

The prevalence we found (16.0%) was similar to the results reported in previous studies in Burkina Faso (14.8% in rural and urban women in 2010 [51] and 19.9% in 2016 [52]). The weighted prevalence of 14.8% was considered to be the greatest among the 33 SSA countries investigated, following the prevalences in Senegal (20.8% in 2011) and Gambia (15.7% in 2013) [51]. Moreover, the high prevalence of 31.0% found by Ramsey et al. in rural Burkina Faso was the highest among the three rural African areas involved in their study [53]. SLT use was associated with undernourishment (aOR = 1.78; *p* < 0.01), as it was in rural Ghanaian women (aOR = 2.78; *p* = 0.002) [54]. Tobacco delivers nicotine, which is an anti-appetite component [55]. There is low food availability in rural Burkina Faso [56], and hunger and an empty stomach generate discomfort. The belief that minimum food intake combined with SLT consumption helps allay hunger further exposes individuals to insufficient food intake. Similar habits were reported for addictions to tea and SLT among Malian Tuaregs living in Sahelian areas and suffering from hunger in a harsh climate [57].

### No significant impairment in the lipid profile or blood sugar among SLT users

Lower HDL cholesterol and higher total cholesterol among smokers and tobacco chewers than among nonusers have been reported [58, 59]. However, the SLT users and nonusers in our current sample had identical mean values of HDL cholesterol and total cholesterol, and there was no significant risk of lipid profile impairment in SLT users (Tables 2 & 3). These results were not surprising in our context because SLT users were more frequently affected by undernourishment (28.6% vs. 13.9% in nonusers; *p* < 0.001) and not by overweight/obesity (only 5.5% vs. 14.4% in nonusers; *p* < 0.001; Table 2). The women in our study would frequently have moderate or insufficient amounts of fat in their bodies, including in blood vessels. Similarly, there was no association with increased blood sugar in users; their mean blood sugar was in fact significantly lower than that of nonusers (3.8 ± 1.5 mmol/l vs. 4.1 ± 1.5 in nonusers; *p* < 0.05; Table 2). However, further investigations should consider the hypothesis of a positive association.

### Sociodemographic factor influences

SLT use increased with age in our study (Table 3). It has previously been reported that elderly individuals have an increased number of cardiovascular risk factors [60] and that SSA countries under demographic and epidemiologic transitions have severely affected cardiovascular risk [61]. An additional modifiable factor, such as SLT use, should be avoided. Uneducated people were frequently exposed to SLT use (Table 3), which indicated that formal instruction, as well as public health education, focused on cardiovascular risk factors and SLT-related health consequences would be effective in reducing prevalence and risk [62].

### Limitations

We used national data from the STEPS survey, which studied the prevalence of and knowledge of common risk factors for noncommunicable diseases. However, the sample size calculation was based on the prevalence of HBP. Data collection methods for dental symptoms were based only on self-reporting and did not include examinations by health professionals. Thus, self-reporting may have included incorrect statements and dental assessments.

## Conclusions

The high prevalence of SLT use in women, unlike ST use, which is nearly non-existent in rural Burkina Faso, indicates a socially permissive attitude towards SLT use by females. The frequent occurrence of dental symptoms among SLT users reinforces the practice and reveals problematic access to oral health care in rural Burkina Faso. The frequent co-use of alcohol can result in the addictive practice known as polyconsumption (of these 2 psychoactive substances), which potentiates the psychological and physical effects and, unfortunately, includes adverse effects. Addictive habits may also include the expectation of immediate hunger suppression in an environment with low food availability. Thus, worsening undernourishment in SLT users might be connected to insufficient food intake resulting from the appetite-suppressing effects of the nicotine delivered by SLT used over the long term. The increases in SBP and differential blood pressure suggest serious cardiovascular concerns associated with SLT use. Furthermore, an overview of the chemical compounds and their levels in locally produced tobacco leaves should be given attention. The lack of increases in waist circumference and total cholesterol may be due to the severity of the undernourishment status of SLT users. Regarding ST use, National Public Health interventions are needed to reduce SLT use and its health-related concerns.

The efficacy of a brief dental office intervention for the general population of smokeless tobacco users was previously described [63]. Nevertheless, dentists and dental assistants should be more available in Burkina Faso. Interventions that could be undertaken may be to identify smokeless tobacco-related oral health problems, offer encouragement to set a quit date for cessation, provide tips on quitting, provide educational videos and encouragement to watch them, provide written educational materials and encouragement to read them, and provide phone call reminders regarding decisions to quit using smokeless tobacco [64].

## Data Availability

The database of the STEPS survey used for this secondary analysis is available at the Ministry of Health of Burkina Faso. It can be requested directed to Dr. Brice Bicaba bicababrico78@gmail.com.

## Abbreviations

aOR: Adjusted odds ratios
BMI: Body mass index
CI: Confidence interval
cOR: Crude odds ratio
DBP: Diastolic blood pressure
HBP: High blood pressure
HDL: High-density lipoprotein
mmHg: Millimetre per mercury
mmol/l: millimole per liter
NCDs: Non-communicable diseases
OR: Odds ratio
SBP: Systolic blood pressure
SLT: Smokeless tobacco
SSA: Sub-Saharan-African
ST: Smoked tobacco
STEPS: STEPwise approach to Surveillance
WHO: World Health Organization
